# Mutation in the *pssA* Gene Involved in Exopolysaccharide Synthesis Leads to Several Physiological and Symbiotic Defects in *Rhizobium leguminosarum* bv. *trifolii*

**DOI:** 10.3390/ijms141223711

**Published:** 2013-12-05

**Authors:** Monika Janczarek, Kamila Rachwał

**Affiliations:** Department of Genetics and Microbiology, Institute of Microbiology and Biotechnology, Maria Curie-Sklodowska University, Akademicka 19 st., Lublin 20-033, Poland; E-Mail: rachwal.kamila@gmail.com

**Keywords:** *pssA* mutant, exopolysaccharide synthesis, metabolic profile, motility, *Rhizobium leguminosarum* bv. *trifolii*, symbiosis, clover

## Abstract

The symbiotic nitrogen-fixing bacterium *Rhizobium leguminosarum* bv. *trifolii* 24.2 secretes large amounts of acidic exopolysaccharide (EPS), which plays a crucial role in establishment of effective symbiosis with clover. The biosynthesis of this heteropolymer is conducted by a multi-enzymatic complex located in the bacterial inner membrane. PssA protein, responsible for the addition of glucose-1-phosphate to a polyprenyl phosphate carrier, is involved in the first step of EPS synthesis. In this work, we characterize *R. leguminosarum* bv. *trifolii* strain Rt270 containing a mini-Tn*5* transposon insertion located in the 3′-end of the *pssA* gene. It has been established that a mutation in this gene causes a pleiotropic effect in rhizobial cells. This is confirmed by the phenotype of the mutant strain Rt270, which exhibits several physiological and symbiotic defects such as a deficiency in EPS synthesis, decreased motility and utilization of some nutrients, decreased sensitivity to several antibiotics, an altered extracellular protein profile, and failed host plant infection. The data of this study indicate that the protein product of the *pssA* gene is not only involved in EPS synthesis, but also required for proper functioning of *Rhizobium leguminosarum* bv. *trifolii* cells.

## 1. Introduction

*Rhizobium leguminosarum* bv. *trifolii* is a gram-negative bacterium that can exist as either a free-living bacterium or a nitrogen-fixing symbiont inside root nodules of its host plant—clover (*Trifolium pratense*). This symbiosis is a complex process requiring the exchange of signaling molecules between both partners. Host plant roots secrete flavonoids that induce the bacterium to produce the Nod factor, the primary determinant for formation of nodules, *i.e.*, specialized new structures within which nitrogen fixation occurs [1]. In response to specific flavonoids, the bacterium synthesizes the Nod factor. After infection of nodule cells, bacteria differentiate into symbiotic forms called bacteroids, which reduce atmospheric nitrogen to ammonia next utilized by the host plant [2]. In addition to the Nod factor, bacteria produce large amounts of acidic extracellular polysaccharide (EPS) required for initiation and elongation of infection threads, *i.e.*, specific tubular structures through which bacteria invade the host plant [3,4]. EPS plays a crucial role in symbiotic interactions with legumes forming indeterminate-type nodules (e.g., *Trifolium*, *Viciae*, *Pisum*, and *Medicago* spp.). Moreover, this polysaccharide contributes to several other processes in free-living rhizobia, such as protection against environmental stresses, nutrient gathering, and attachment to both abiotic surfaces and host plant roots, which ensure adaptation to changing soil conditions [5]. EPS-deficient mutants of *R. leguminosarum* bvs. *trifolii* and *viciae*, and *Sinorhizobium meliloti* induce small, only partially infected, nodule-like structures on roots of their host plants that are ineffective in nitrogen fixation [3,4,6]. On the other hand, EPS-overproducing *R. leguminosarum* bv. *trifolii* strains display significantly enhanced competitiveness, nodulation ability, and symbiotic efficiency [7]. In contrast, mutant strains of *R. leguminosarum* bv. *phaseoli* defective in EPS production induce nitrogen-fixing nodules on *Phaseolus* plants, which form determinate-type nodules [8].

EPS produced by *R. leguminosarum* is a polymer composed of octasaccharide repeating units which contain d-glucose, d-glucuronic acid, and d-galactose residues in a molar ratio 5:2:1 substituted with *O*-acetyl and pyruvyl groups [5,9]. Up to now, the knowledge of the genetic control of EPS synthesis in *R. leguminosarum* is fragmentary known and functions of only a few proteins have been experimentally confirmed. The first step of EPS synthesis is conducted by a glucose-IP-transferase encoded by the *pssA* gene, which transfers glucose-1-phosphate from UDP-glucose to a C_55_-isoprenylphosphate (IP) carrier located in the inner membrane [10,11]. The successive steps of EPS synthesis are conducted by protein products of *pss* genes grouped in a large chromosomal EPS cluster I [12]; glucuronosyl-(β1–4)-glucosyl transferase PssDE and glucuronosyl-(β1–4)-glucuronosyl transferase PssC catalyze the second and the third step of the unit synthesis, respectively [10]. Moreover, other *pss* genes of this cluster are assumed to be engaged in the next steps of the synthesis and modification of EPS (*pssGHI* and *pssRMK* genes, respectively). Among them, a ketal pyruvate transferase encoded by *pssM* proved to be responsible for addition of the pyruvyl group to the subterminal glucose in the repeating unit [13]. However, enzymes participating in the remaining steps of the unit synthesis have not been identified yet.

Biosynthesis of EPS in rhizobia is a complex process regulated at both transcriptional and posttranslational levels and influenced by various environmental factors [5]. However, only a few proteins involved in regulation of EPS synthesis have been identified in *R. leguminosarum* so far, among them PsiA and PsrA encoded by genes located on the symbiotic megaplasmid of *R. leguminosarum* bv. *phaseoli* [14–16], ExoR of *R. leguminosarum* bv. *viciae* [17] and PssB of *R. leguminosarum* bvs. *trifolii* and *viciae* [18,19] responsible for negative regulation, and RosR of *R. leguminosarum* bv*. trifolii* being a positive regulator of this process [20,21]. The RosR protein belongs to a family of Ros/MucR transcriptional regulators, which contain a characteristic Cys_2_His_2_ type zinc-finger motif and are involved in regulation of EPS synthesis in rhizobial species [20]. The mutation in *R. leguminosarum* bv. *trifolii rosR* resulted in a substantial decrease in EPS production and symbiotic defects [21].

A mutation in the *psiA* gene does not affect EPS production, but additional copies of this gene inhibit the synthesis of this polymer. The effect of multiple *psiA* copies is overcome in the presence of additional copies of *psrA* or *pssA*, indicating that a balanced copy number of these genes is indispensable for proper EPS synthesis [14,15,22]. In addition, ExoR influences EPS synthesis in *R. leguminosarum*, as a mutant in the *exoR* gene produces higher amounts of EPS than the wild-type strain [17].

In this work, we report that a mutation in the 3′-end of the *pssA* gene affects several physiological and symbiotic properties of *R. leguminosarum* bv. *trifolii*, changing the adaptation ability of this bacterium to live both in the free stage and in association with the host plant. In addition, the influence of the *exoR* gene on *pssA* expression and EPS production was studied.

## 2. Results and Discussion

### 2.1. Mutagenesis of the 3′-End of *pssA* and the Influence of This Mutation on Exopolysaccharide Production and Symbiosis with Clover

**Figure 1.**
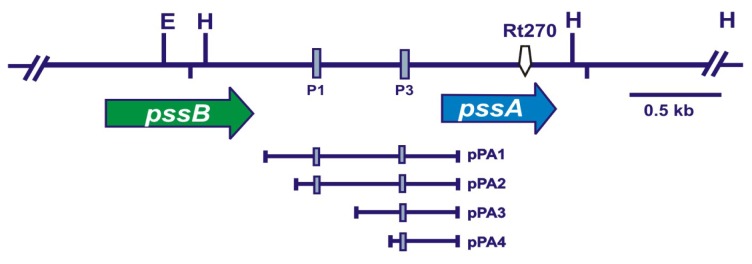
Physical and genetic map of the genomic region of *Rhizobium leguminosarum* bv. *trifolii* 24.2 carrying *pssB* and *pssA* genes. The green and blue arrows below the map show the direction of transcription of *pssB* and *pssA*, respectively. E, *Eco*RI, H, *Hin*dIII. P1 and P3 are promoter sequences for the *pssA* gene. Lines below the arrows indicate fragments of the *pssA* regulatory region cloned upstream of *lacZ*, which are present in individual *pssA-lacZ* transcriptional fusions pPA1-pPA4.

Previous studies of other researchers indicated that the *pssA* gene encodes a protein of the length of 200 amino acids, which is located in the bacterial inner membrane and involved in the first step of EPS synthesis [7,11,14]. In addition, our earlier data showed that the *pssA* gene represents an individual open reading frame located downstream of *pssB* and is present in genomes of all strains belonging to three *R. leguminosarum* biovars (*trifolii*, *viciae* and *phaseoli*) (Figure 1) [23]. In this work, we describe several physiological defects in *R. leguminosarum* bv. *trifolii* strain 24.2 caused by a mutation in the 3′-end of the *pssA* gene, which encodes the catalytic domain of the enzyme.

Using a random mini-Tn*5* mutagenesis, several derivatives of *R. leguminosarum* bv. *trifolii* 24.2 were obtained, including a mutant strain named Rt270 with a transposon inserted in a position between 412 and 413 nt of a 600-nt long coding region of *pssA*. This mutant formed characteristic small nonmucoid colonies on agar plates differing significantly from those formed by the wild-type strain. Quantitative EPS assays indicated that the mutant strain Rt270 did not produce any amounts of this polysaccharide (Rt270 = 0, whereas Rt24.2 = 1426 ± 169 mg L^−1^). These data confirm that the mutation located in the *pssA* 3′-end encoding the *C*-terminal domain of the protein totally abolishes its enzymatic function. Previous studies of other researchers indicated that the *N*-terminus of PssA is hydrophobic, that suggested its function in the interaction with the bacterial inner membrane, whereas the *C*-terminus of this protein is more hydrophilic and responsible for the enzymatic activity [14,24]. Mutations located in both the regulatory region and the upper part of the coding region of *pssA* yielded similar negative effects on EPS production (lack of EPS synthesis) in *R. leguminosarum* bvs. *trifolii*, *viciae* and *phaseoli* [6,7,25–27]. These data indicate that the PssA protein plays a key role in this biosynthetic pathway and an appropriate level of *pssA* expression is required for proper production of EPS.

Moreover, the mutation located in the *pssA* 3′-end significantly affected the symbiotic properties of the Rt24.2 strain, similarly as it was previously evidenced for other *pssA* mutants of *R. leguminosarum* bvs. *trifolii* and *viciae* [6,7,25,27]. The Rt270 mutant elicited about a three-fold lower number of nodules on clover plants than the wild-type strain (3.7 ± 2 * in comparison to 12.4 ± 3 nodules induced by Rt24.2), which were ineffective in nitrogen fixation (shoot fresh weight - 34.1 ± 6.0 * mg plant^−1^ in comparison to 63.4 ± 10.1 for Rt24.2, *p* values < 0.05; Student’s *t* test).

**Figure 2.**
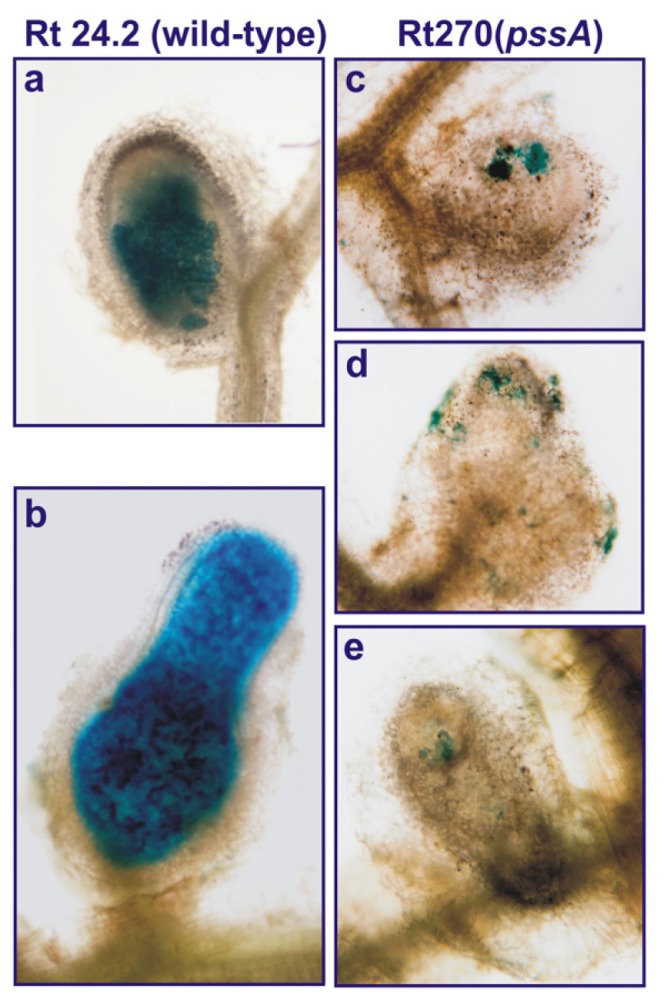
Light microscopy of nodules induced on roots of clover plants (*Trifolium pratense*) by the *Rhizobium leguminosarum* bv*. trifolii* wild-type strain 24.2 and the *pssA* mutant Rt270 harboring pJBA21Tc plasmid with *gusA* reporter gene for β-glucuronidase. (**a**,**b**) Rt24.2 wild-type nodules at 7 and 21 days post infection, respectively; (**c**–**e**) Rt270 nodules at 7 (**c**) and (**d**), and 21 days post infection (**e**). The nodules were stained for GUS activity.

In addition, nodule colonization by the Rt270 strain was drastically decreased. A great majority of nodules induced by this mutant were not occupied by the bacteria (data not shown), and in sporadic nodules, only single plant cells were infected (Figure 2). It is well known that the presence of EPS surrounding bacterial cells is indispensable for these rhizobial species which establish symbioses with legumes forming indeterminate-type nodules [5,27]. Similarly, in *R. leguminosarum* bv*. viciae*, which infects *Pisum sativum*, *Vicia faba* and *Vicia sativa* plants also forming indeterminate-type nodules, a mutation in *pss4* homologous to *R. leguminosarum* bv. *trifolii pssA* resulted in inhibition of EPS synthesis and ineffective symbiosis with these host plants [6,25].

### 2.2. Mutation in *pssA* Affects Bacterial Motility

The ability of rhizobia to migrate is very important for their competitiveness and infection of host plant roots, which both are essential for establishment of effective symbiosis. Therefore, we decided to establish whether the mutation in *pssA* affects the motility of *R. leguminosarum* bv*. trifolii*. For this experiment, M1 minimal medium and two rich 79CA and TY media were used, which all contained 0.3% or 0.7% agar. In the case of the wild-type Rt24.2 strain, it was observed that migration of bacteria was dependent on both the agar concentration and the medium used (Table 1). A longer migration distance was observed for media containing 0.3% agar than for those containing 0.7% agar. Moreover, the wild-type bacteria migrated more effectively in the rich media than in the minimal M1 medium. In contrast, the Rt270 strain showed significantly slower migration in relation to the Rt24.2 strain in all the tested media. This negative effect was the most visible in the case of the rich media containing 0.7% agar. These data indicate that the mutation in the *pssA* gene affects the motility of rhizobial cells.

**Figure 3.**
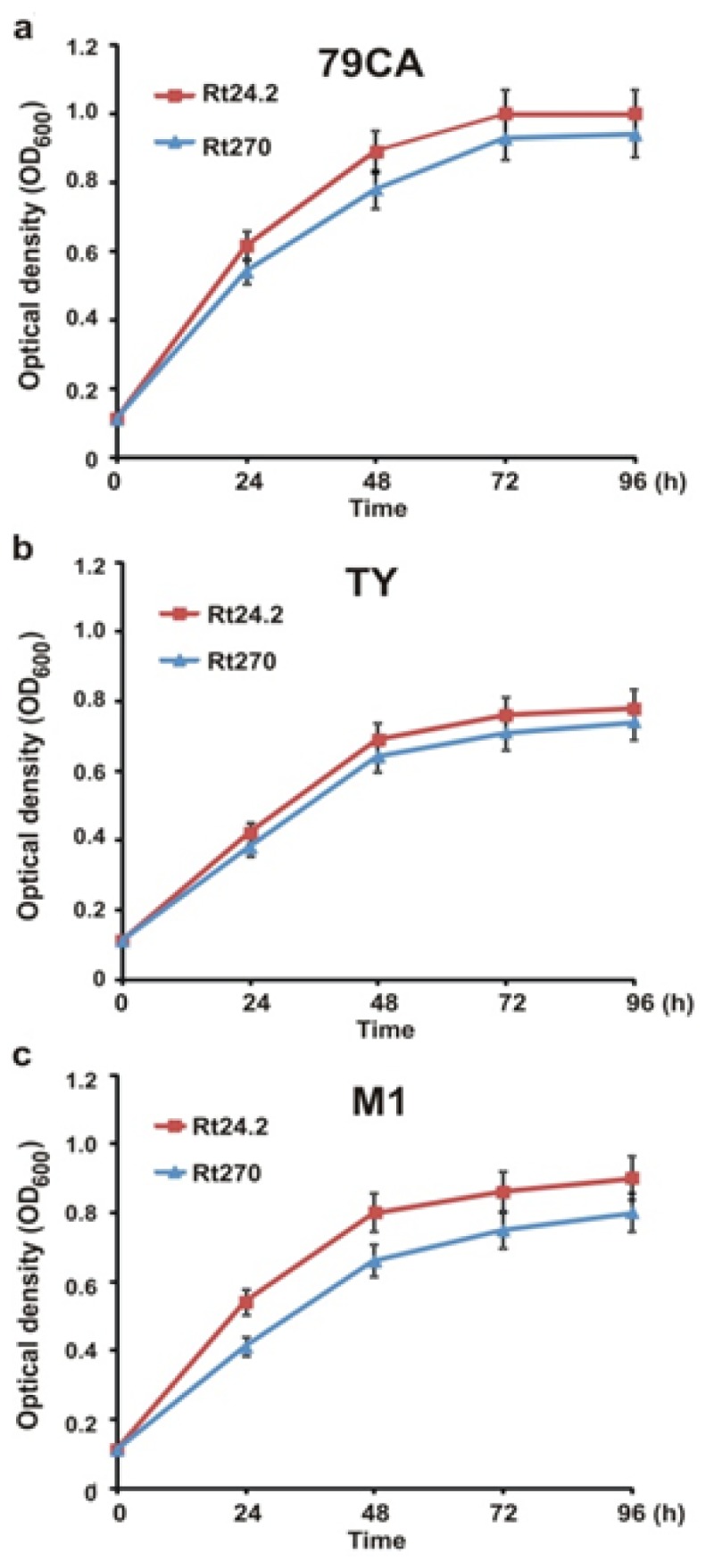
The growth of the *Rhizobium leguminosarum* bv*. trifolii* wild-type Rt24.2 and the *pssA* mutant Rt270 in rich 79CA (**a**) and TY (**b**) media and in minimal M1 medium (**c**).

In order to elucidate if observed differences in migration between the Rt24.2 and Rt270 strains were a result of their different growth under particular conditions, the kinetics of growth of these bacteria was established in M1, 79CA and TY media (Figure 3).

It was observed that the *pssA* mutant grew nearly as effectively as the wild-type strain in both the energy-rich media, whereas its growth rate was moderately lower than that of the parental strain in the minimal medium. These data eliminated growth effectiveness as an essential factor causing differences in the motility of the tested strains, and did not elucidate why the Rt270 mutant showed significantly slower migration in relation to the Rt24.2 strain in all the tested media. Therefore, we decided to establish if the *pssA* mutation affects the expression of motility-related genes in *R. leguminosarum* bv*. trifolii*. To this end, transcriptional fusions of *rem*, *visN* and *flaA* genes with a reporter *gusA* gene were introduced into the Rt24.2 and Rt270 strains, and β-glucuronidase activities were determined after growing these bacteria in the 79CA and M1 media. In the Rt24.2 strain, the expression of all three fusions was much lower in the minimal medium in comparison to the rich medium, that explained the slower migration of the wild-type bacteria under these conditions (Table 2). Moreover, promoter activities of the *visN-gusA*, *rem-gusA*, and *flaA-gusA* fusions were significantly downregulated in the Rt270 mutant in both the tested media, suggesting that the mutation in the *pssA* gene affects the motility of bacterial cells, at least in some part, by modulation of the expression of these motility-related genes.

Up to now, motility has been described in a wide range of bacteria including members of *Vibrio*, *Escherichia*, *Salmonella*, *Proteus*, *Pseudomonas*, *Sinorhizobium*, *Agrobacterium*, and *Rhizobium* [28–32]. The widespread occurrence of this bacterial property suggests that motility plays a significant role in colonization of natural environments by microorganisms. For example, it was indicated that swarming motility in *Proteus mirabilis* facilities ascending colonization of the urinary tract [32] and in *Pseudomonas fluorescens* colonization of alfalfa rhizosphere [33]. However, in nitrogen-fixing bacteria, motility is currently not well characterized. Tambalo and co-workers [34] described that growth on energy-rich media and agar concentration were critical parameters for swarming of two *R. leguminosarum* bv*. viciae* strains 3841 and VF39SM. The results presented in this work also indicate that these parameters influenced swimming motility of the Rt24.2 strain. Moreover, we observed that the mutation in the *pssA* gene significantly affected the motility of *R. leguminosarum* bv*. trifolii* cells. Based on transcriptional fusion and growth experiments, it was found that this defect was mainly caused by a decreased expression of the *visN*, *rem* and *flaA* genes in the mutant strain. VisN and VisR are LuxR-type global regulators of flagellar, motility, and chemotaxis genes in *R. leguminosarum* and *S. meliloti* [35,36]. VisN/R upregulates the expression of *rem*, and Rem positively affects the expression of *flaA* and other genes involved in flagellum formation, motility, and chemotaxis [35–37]. In *S. meliloti*, CbrA and two ExoR/ExoS/ChvI and quorum sensing ExpR/SinR/SinI regulatory systems control *visN* and *visR* expression [5,28,38]. In addition, MucR, which is a regulator of *nodD*, *fix*, and succinoglycan and galactoglucan synthesis genes in this bacterium, negatively regulates the transcription of *rem*. All these data indicate occurrence of a complex regulatory network in rhizobia linking EPS production, motility, and quorum sensing [5]. In *R. leguminosarum*, a MucR homolog, RosR, and ChvG (ExoS) histidine kinase of a two-component signal transduction system were found to positively affect *pssA* expression [20,26,39]. A mutation in *chvG* caused a number of pleiotropic phenotypes including inability to grow on proline, glutamate, histidine, or arginine as the sole carbon source, synthesis of smaller amounts of acidic and neutral surface polysaccharides, destabilization of the outer membrane, and symbiotic defects on peas, lentils, and vetch [39]. Recently, it has been reported that a mutant strain of *R. leguminosarum* bv*. viciae* 3841, in which lipopolysaccharide (LPS) does not contain 27-hydroxyoctacosanoic acid modification, is impaired in motility and biofilm formation [40]. In this strain, the expression of *visN*, *rem*, and *flaA* genes was also significantly decreased, suggesting that the mutation affects gene expression at the highest levels of regulation, similarly as in the case of the *pssA* mutant. Likewise, a mutation in the *S. meliloti fadD* gene involved in fatty acid metabolism affects cell migration and nodulation efficiency on alfalfa roots [30]. These data indicate that the presence of extracellular polysaccharides such as EPS and LPS and their proper modifications are important for motility of rhizobia.

### 2.3. Phenotype Analysis of the *pssA* Mutant Using Biolog Tests

In addition, we decided to establish whether a mutation in the *pssA* gene affects metabolic capability of *R. leguminosarum* bv. *trifolii*. In order to define the phenotype profile of the *pssA* mutant Rt270 in relation to the wild-type strain Rt24.2, the PM system (Biolog) was used. PM1, PM2A, PM3B, and PM4A plates were chosen for examination of utilization of 190 carbon, 95 nitrogen, 59 phosphorus, and 35 sulphur sources, respectively. In addition, PM9 plates were used to examine the growth in the presence of various stress factors.

**Figure 4.**
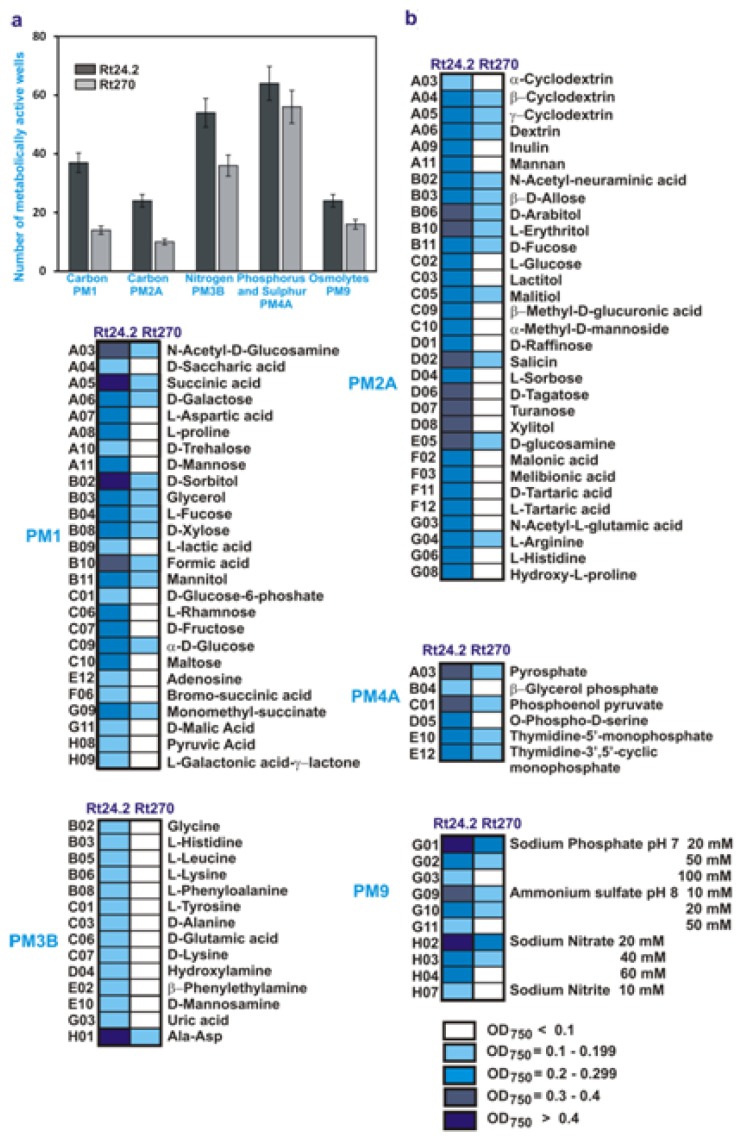
(**a**) A quantitative and qualitative comparison of the carbon, nitrogen, phosphorus and sulphur sources utilized by the *pssA* mutant Rt270 and the wild-type strain Rt24.2; and (**b**) Metabolic differences determined between the Rt24.2 and Rt270 strains using PM plates. Data shown are means of three independent experiments.

In general, the *pssA* mutant utilized fewer energy sources than the parental strain (Figure 4a,b). The major differences were observed in carbon source utilization (PM1 and PM2A plates). The Rt24.2 strain used 60 carbon sources, whereas the Rt270 mutant only 28 nutrients. Moreover, this mutant utilized many of these carbon sources less efficiently than the wild-type bacteria. For example, succinic acid and sorbitol were two of the best-utilized carbon sources by the Rt24.2 strain, whereas they were less effectively utilized by the *pssA* mutant. Likewise, 14 nitrogen sources were less efficiently used by the Rt270 mutant than by the parental strain (plate PM3B). With regard to utilization of phosphorus and sulphur sources, the *pssA* mutant did not differ essentially from the wild-type strain (differences were observed only for six compounds) (plate PM4B).

Subsequently, the sensitivity of the *pssA* mutant to several osmolytes was tested using PM9 plates. Mutant Rt270 exhibited increased sensitivity to such compounds as Na_3_PO_4_, (NH_4_)_2_SO_4_, and NaNO_3_. In contrast to the wild-type Rt24.2, Rt270 did not survive in 100 mM Na_3_PO_4_, 50 mM (NH_4_)_2_SO_4_, 60 mM NaNO_3_, and 10 mM NaNO_2_ (Figure 4b).

In summary, it was indicated that the *pssA* mutant was impaired in its ability to utilize several carbon compounds and exhibited an increased sensitivity to some osmolytes, suggesting that the lack of the functional PssA protein affects metabolic activities in *R. leguminosarum* bv. *trifolii* cells.

This phenomenon could be, to some extent, explained by the data from proteomic analyses of cellular proteins described by Guerreiro and others [41], who reported that the *pssA* mutation significantly affected the synthesis levels of 22 proteins in *R. leguminosarum* bv. *trifolii* ANU437. These researchers identified only two proteins from this *pssA* mutant stimulon as a glutamine-binding periplasmic protein and MigA homologue involved in the synthesis of LPS or EPS in *P. aeruginosa*, respectively. Bioinformatic analyses of the *N*-end sequences of the proteins reported by Guerreiro *et al.* [41] against protein sequences of *R. leguminosarum* strains available in databases allowed us to identify the remaining proteins as a putative NADH-dependent FMN reductase (spots n4 and n6), a FMN reductase (n5 and n7), a putative taurine catabolism dioxygenase (n8), a DSBA oxidoreductase/putative outer membrane protein (n16), a putative outer membrane lipoprotein/putative ABC transporter substrate-binding protein (n17), a periplasmic component of the ABC-type sugar transport system (n18), a periplasmic component of the ABC-type nitrate/sulphonate/bicarbonate transport system (n19), and a protein from the formate/nitrite transporter family (n20). In contrast, the protein synthesis pattern of the *R. leguminosarum* bv. *viciae pssC* mutant producing three-fold less EPS than the wild-type strain showed no differences from that of the parental strain, whereas the EPS-deficient *pssD* and *pssE* mutants had alterations in only eight proteins, which were included into the 22 changes found in the *pssA* mutant [41]. Among these *pss* mutants of *R. leguminosarum*, such large protein profile alterations proved to be unique for the *pssA* mutation. These data indicate that, the PssA protein, being the component of the enzymatic complex located in the inner membrane, might serve additional function(s) in membrane stability, and thus in metabolic processes. Previously, it was found that the *pssA* gene present in additional copies restored the mucoid phenotype of several EPS-deficient mutants of *R. leguminosarum* bv. *trifolii*, which have mutations in genes not directly involved in EPS synthesis, suggesting possible relation(s) between EPS production and metabolic pathways [24]. Also, a mutation in *chvG* caused pleiotropic effects in *R. leguminosarum* bv. *viciae*, among them inability to grow on several carbon sources (proline, glutamate, histidine, and arginine) [39]. Similarly, for *S. meliloti exoS* and *chvI*, it has been reported recently that mutations in these genes affect utilization of over 21 different carbon sources [42,43]. All these data suggest existence of a complex interconnection of EPS biosynthesis with other metabolic pathways in rhizobia.

### 2.4. The Influence of *pssA* Mutation on the Profile of Extracellular Proteins

**Figure 5.**
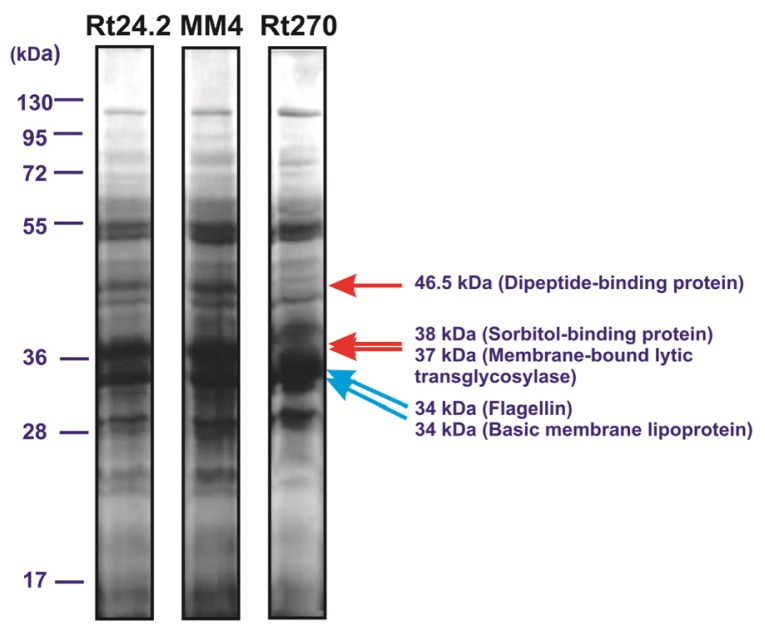
Extracellular protein profiles of the *R. leguminosarum* bv. *trifolii* wild-type strain 24.2 and two EPS-deficient Rt270 and MM4 mutants. The migration positions of molecular mass markers are shown. Individual slot contains 15 μg of the extracellular protein fraction. Protein bands missing or of decreased amounts in the *pssA* mutant profile are marked by red arrows, whereas proteins of higher amounts in relation to the wild-type profile are marked by blue arrows.

Proteins secreted by rhizobia play an important role in both nutrient uptake and infection of host plant roots. The differences observed in the utilization of several carbon sources between the *pssA* mutant and the wild-type strain prompted us to compare the extracellular protein profiles of these strains, because this protein fraction of the *pssA* mutant was not included into the proteomic analyses performed by Guerreiro and others [41]. To this end, proteins from culture supernatants of the Rt24.2 and the Rt270 strains were isolated and analyzed in SDS-PAGE (Figure 5).

The profile of extracellular proteins of *R. leguminosarum* was established previously by Krehenbrink and Downie and its particular proteins were identified [44]. The comparison of extracellular protein profiles of the Rt24.2 and Rt270 strains indicated that the fraction of the *pssA* mutant differed from that of the wild-type bacteria; Rt270 secreted higher amounts of some proteins, whereas others were absent. A protein of molecular weight of ~46.5 kDa and a predicted function of dipeptide-binding protein was almost not present in the fraction of this mutant. Additionally, proteins of 38 and 37 kDa and a function of sorbitol-binding protein and a membrane-bound lytic transglycosylase, respectively, were not detected in the extracellular protein fraction of this mutant. In contrast, two proteins of molecular masses 34-kDa and a function of flagellin and a basic membrane lipoprotein, respectively, were found in higher amounts in comparison to those present in the fraction of the wild-type strain. The differences observed between the extracellular protein fractions of the *pssA* mutant and the wild-type strain suggest disturbances in secretion of these proteins. Another EPS-deficient mutant MM4 of *R. leguminosarum* bv. *trifolii* was included in this analysis as a control, which showed no differences in its extracellular protein pattern in relation to that of the parental strain. This suggested that the changes observed in the protein profile of the *pssA* mutant were not a result of the lack of EPS.

In *R. leguminosarum*, several proteins are secreted to extracellular space, among them proteins involved in modification of EPS (PlyA and PlyB glycosyl hydrolases), motility (flagellar hook, flagellin), surface attachment (cadherin-like proteins, adhering protein RapA2), and nutrient uptake (dipeptide-binding protein, sorbitol-binding protein, glycosyl hydrolase, Leu/Ile/Val-binding protein BraC, sugar-binding protein, ribose-binding protein, arginine/ornithine-binding protein, peptidyl prolyl *cis*-*trans* isomerase, and nucleoside diphosphate kinase) [44,45]. A majority of these proteins are secreted by the type I PrsDE system, which transports proteins of widely varied size and predicted function from the cytoplasm across both membranes to the extracellular space. This is in contrast to many Type I systems from other microorganisms that typically secrete specific substrates encoded by genes often localized in close proximity to the genes encoding the secretion system itself [46,47].

### 2.5. Sensitivity of the Wild-Type and *pssA* Mutant Strains to Detergents, Ethanol and Antibiotics

To characterize further the *pssA* mutant, sensitivity assays to detergents, ethanol, and antibiotics were performed, which provide indirect evidence for disturbances in membrane integrity and/or functioning.

Two *R. leguminosarum* bv. *trifolii* mutant strains MM3 and MM4 deficient in EPS production were additionally included in this analysis (Table 3). The mutant Rt270 was found to be more sensitive to sodium deoxycholate (DOC) and ethanol, and slightly more sensitive to sodium dodecyl sulfate (SDS) than the parental strain. The MM3 and MM4 mutants showed very similar sensitivity profiles to each other, and they were only slightly more sensitive to SDS, DOC, and sarcosyl, in relation to the wild-type bacteria. In comparison to the phenotypes of these mutants, the Rt270 strain was significantly more sensitive to DOC and ethanol. These data indicate that, although the presence of EPS is important to protect rhizobial cells against stress factors, the Rt270 mutant showed the highest sensitivity to the tested stressors among the Exo^−^ mutants analyzed. Based on these data, we conclude that the mutation in *pssA* in some part affects the integrity of the rhizobial outer membrane. In addition, increased sensitivity to several membrane stressors (DOC, SDS, and sarcosyl) was found in other mutant strains of *R. leguminosarum*, which have mutations in genes involved in the synthesis of components of the outer membrane. These include the *fabF2/F1* mutant deficient in 27-hydroxyoctacosanoate-modified LPS, the *ropB* mutant lacking outer membrane protein, and the *pssP* mutant unable to transport EPS [39,40,48,49].

In addition, sensitivity of the *pssA* mutant to several antibiotics was determined (Table 4). Unexpectedly, the Rt270 mutant proved to be more resistant to a majority of the tested antibiotics (with the exception of gentamicin and tetracycline). The most remarkable differences were observed for 6 antibiotics (ampicillin, penicillin G, amoxicillin, bacitracin, oxocillin, and polimyxin B), to which the *pssA* mutant was totally resistant, in contrast to the wild-type bacteria. The Rt270 mutant was also less sensitive to erythromycin, chloramphenicol, and neomycin.

However, the antibiotic profiles of both the MM3 and MM4 mutants did not differ significantly from that of the wild-type strain; they were slightly more sensitive to gentamicin, tetracycline, amoxicillin, and ampicillin. The changes observed in the sensitivity profile of the *pssA* mutant in relation to the parental strain concerned antibiotics with various mechanisms of action in bacterial cells. This suggested that the outer membrane of this strain, which has a critical role as a protective barrier against harmful substances, became less permeable to these antibiotics. Another possibility is alteration in functioning of non-specific porin channels in the outer membrane, which are used by some antibiotics (e.g., chloramphenicol) or more effective transport of these substances outside bacterial cells [50]. The antibiotic sensitivity profile of the *pssA* mutant also differs from those described for other mutants of *R. leguminosarum* bv. *trifolii* impaired in EPS synthesis (*pssP* and *rosR*), indicating that the phenotype observed is unique for this mutation [21,48]. On the other hand, increased sensitivity to erythromycin and Polimyxin B, confirming destabilization of the outer membrane, was reported in two *chvG* and *rpoB* mutants of *R. leguminosarum* bv. *viciae* [39].

### 2.6. ExoR Negatively Affects the Expression of the *pssA* Gene and Exopolysaccharide Production

In *S. meliloti*, EPS production was found to be affected by several environmental factors, including phosphate, nitrogen, and catabolite repression [5]. In addition, in *R. leguminosarum* bv. *trifolii*, phosphate and catabolite repression influenced EPS synthesis. However, up to now, a role of nitrogen in modulation of EPS production was not studied in this bacterium. Therefore, we decided to establish whether this environmental signal also affects *pssA* expression and EPS production in *R. leguminosarum* bv. *trifolii*, and in addition, if the ExoR regulator might be engaged in this regulation. In *S. meliloti*, ExoR regulates the expression of the *exoYFQ* operon, which is involved in the biosynthesis of exopolysaccharide named succinoglycan (EPS I) [51,52]. A protein product encoded by the *exoY* gene is homologous to *R. leguminosarum* PssA. In order to establish the influence of *exoR* on *pssA* expression and EPS production in *R. leguminosarum* bv. *trifolii*, a DNA fragment encompassing the *exoR* gene from this bacterium was amplified in PCR and sequenced. Bioinformatic analyses indicated that Rt24.2 *exoR* encoded a protein of the length of 267 aa, which is highly homologous to ExoR of *R. leguminosarum* bv. *viciae* (98% identity, 100% similarity) and ExoR of *Rhizobium etli* (94% identity, 96% similarity), and shows a lower similarity to ExoR of *S. meliloti* (69% identity, 82% similarity). Subsequently, Rt24.2 *exoR* was cloned into a high-copy-number vector pBBR1MCS-5, which is present in six to seven copies in *R. leguminosarum* cells [7], and the pRR1 plasmid obtained was introduced into the Rt24.2 strain and its derivatives. Then, the influence of multiple copies of this gene on *pssA* expression and EPS production under different growth conditions was studied (Table 5).

In this experiment, four *pssA-lacZ* transcriptional fusions (pPA1-pPA4) containing different fragments of the *pssA* upstream region with two P1 and P3 functional promoters were used (Figure 1, Table 5). In order to exclude the influence of the empty pBBR1MCS-5 vector on transcriptional activity of the tested fusions, this plasmid was introduced into the Rt24.2 derivatives harbouring the pPA1-pPA4 plasmids, and β-galactosidase assays were performed. It was established that the pBBR1MCS-5 vector present in the Rt24.2 derivatives did not affect significantly the transcriptional activity of the tested fusions. In the case of the Rt24.2 strain harboring the pPA1-pPA4 plasmids, slightly lower *pssA-lacZ* expression was observed under high-ammonium (10 mM NH_4_Cl) than low-ammonium (0.1 mM NH_4_Cl) conditions for all the transcriptional fusions analyzed. Moreover, the introduction of multiple *exoR* copies into the Rt24.2 (pPA1-pPA4) derivatives resulted in a high decrease in *pssA-lacZ* expression, and this effect was significantly stronger under the low-ammonium conditions. A similar tendency was observed for all the tested fusions: a 1.86-fold (pPA2 and pPA4) to a 2.96-fold (pPA1) decrease in *pssA-lacZ* expression in ammonium deficiency, when compared between the Rt24.2 and Rt24.2 (pRR1) strains. Therefore, we conclude that the regulatory region with the P3 promoter present in the pPA4 fusion is responsible and sufficient for negative regulation of the *pssA* expression by ExoR.

Regarding the EPS production, it was observed that the Rt24.2 strain synthesized slightly more EPS under high- than low-ammonium conditions (960 ± 61 and 745 ± 59 mg L^−1^, respectively). The presence of multiple *exoR* copies resulted in a decrease in EPS synthesis, especially under ammonium deficiency. The Rt24.2(pRR1) strain produced 404 ± 45 mg EPS L^−1^ in the presence of 0.1 mM NH_4_Cl and 672 ± 53 mg L^−1^ in the presence of 10 mM of this nutrient. The Rt24.2 derivative harbouring the pBBR1MCS-5 vector produced similar amounts of EPS under high- and low-ammonium conditions (942 ± 57 and 732 ± 62 mg L^−1^, respectively) as those secreted by the wild-type strain.

In summary, the data presented in this work indicate that ExoR negatively affects in some way (directly or indirectly) *pssA* expression and EPS production in *R. leguminosarum* bv. *trifolii* and this effect is more pronounced under low-ammonium conditions. Similarly, Reeve and associates [17] indicated that *R. leguminosarum* bv. *viciae* strain WSM710 produced significantly less EPS in the absence of NH_4_Cl than in the presence of this nitrogen source, and confirmed a role of *exoR* in this negative regulation. An *exoR* mutant of this strain produced three-fold more EPS than the wild-type in the minimal medium devoid of the nitrogen source. The ExoR proteins of *R. leguminosarum* bvs. *trifolii* and *viciae* show similarity to the ExoR regulator of *S. meliloti*, which negatively affects expression of several *exo* genes involved in EPS I synthesis, among them the *exoYFQ* operon [51–53]. As in the case of the *exoY* gene, the transcription of *pssA* in *R. leguminosarum* bv. *trifolii* is directed from two P1 and P3 promoters [26,54] and is regulated by ExoR and ammonium concentration. It has been evidenced that the *S. meliloti* ExoR protein is located in the periplasmic space and acts together with the two-component ExoS/ChvI regulatory system affecting the expression of both EPS I and flagellum biosynthesis genes [55,56]. An *exoR* mutant overproduces succinoglycan and is symbiotically defective, indicating that a proper level of EPS synthesis is indispensable for effective symbiosis of *S. meliloti* with alfalfa [57,58], likewise in symbiotic interactions of *R. leguminosarum* bvs. *trifolii* and *viciae* with their respective host plants [8].

## 3. Experimental Section

### 3.1. Bacterial Strains, Plasmids and Growth Conditions

The bacterial strains, plasmids and oligonucleotide primers used in this study are listed in Table 6. *R. leguminosarum* bv. *trifolii* strain 24.2 and its derivatives were grown in 79CA medium with 1% glycerol as a carbon source [59], TY (tryptone-yeast medium), and minimal M1 medium [60] with 1% glycerol and 2 mL L^−1^ stock solution of Dilworth’s vitamin [61] at 28 °C. *E. coli* strains were cultured in Luria-Bertani (LB) medium at 37 °C [60]. To establish the influence of ammonium on the expression of *pssA-lacZ* fusions, Rt24.2 derivatives were grown in M1 medium containing 0.1 mM (low-ammonium conditions) or 10 mM NH_4_Cl (high-ammonium conditions). When required, antibiotics were used at the following final concentrations: kanamycin, 40 μg mL^−1^; gentamicin, 10 μg mL^−1^; spectinomycin, 40 μg mL^−1^; ampicillin, 100 μg mL^−1^; tetracycline, 10 μg mL^−1^; and rifampicin, 40 μg mL^−1^. In some experiments, EPS-deficient MM3 and MM4 mutant strains were used, which were obtained after random mutagenesis of the Rt24.2 strain using the mTn*5*SS*gusA*40 transposon [62].

### 3.2. DNA Methods

Standard techniques were used for genomic and plasmid DNA isolation, restriction enzyme digestion, electrophoresis, cloning, and transformation [60]. For PCR amplifications, Ready *Taq* PCR Reaction Mix (Sigma-Aldrich, St. Louis, MO, USA) was used. DNA fragments and plasmid constructs were sequenced using the BigDye terminator cycle sequencing kit (Applied Biosystems, Foster City, CA, USA) and the ABI Prism 310 sequencer. Database searches were done with the FASTA and BLAST programs (http://www.ebi.ac.uk/fasta33/ [67]) from the National Center for Biotechnology Information (Bethesda, MD, USA) and European Bioinformatic Institute (Hinxton, UK).

### 3.3. Mutagenesis of the *pssA* Gene

For mutagenesis of the 3′-end of the *pssA* gene, an EZ::TN™<KAN-2> Insertion Kit (Epicenter Technology, Madison, WI, USA) was used, which enables generation of random mini-Tn*5* transposon insertions into target DNA. For this experiment, a pM34 plasmid carrying the entire *pssA* gene was used as a target. Tn*5* locations in pM34 derivatives were confirmed by restriction and sequencing analyses. Among the plasmids obtained, pMT27 with a Tn*5* insertion located between 412 and 413 bp of the *pssA* coding region was chosen for further studies (Figure 1, acc. no. AF316883). A 4.6-kb *Eco*RI fragment of this plasmid was cloned into the pSUP202 vector, yielding pMSUP27. This construct was introduced into the *E. coli* S17-1 strain by transformation, and subsequently into the Rt24.2 strain via biparental conjugation. As a result, a clone named Rt270, which formed nonmucoid colonies on plates containing 79CA medium supplemented with kanamycin and rifampicin, was isolated.

### 3.4. Cloning of the *exoR* Gene into the pBBR1MCS-5 Plasmid

Based on the genomic sequence of *R. leguminosarum* bv. *viciae* 3841 [68], pExoR1 and pExoR3 primers were designed for amplification of *exoR* from the Rt24.2 strain. Using this primer pair, a 1.3-kb fragment containing the entire *exoR* gene was amplified. After digestion with *Eco*RI restriction enzyme, the PCR product obtained was cloned into the pUC19 vector, yielding pEXOR13. The insert of this plasmid was sequenced and the sequence obtained was deposited in the GenBank database (acc. no. DQ358643). Subsequently, the 1.3-kb *Eco*RI fragment was inserted into the respective site of the pBBR1MCS-5 vector, yielding the pRR1 plasmid.

### 3.5. The Influence of Multiple *exoR* Copies on *pssA* Expression and EPS Production

To study the effect of multiple *exoR* copies on *pssA* transcription and EPS synthesis, the pRR1 and pBBR1MCS-5 plasmids were introduced by conjugation into Rt24.2 (pPA1)-Rt24.2 (pPA4) strains carrying transcriptional *pssA-lacZ* fusions. Afterwards, bacteria were grown on 79CA agar plates supplemented with gentamicin and tetracycline. For determination of β-galactosidase activity, Rt24.2 derivatives carrying pPA1-pPA4 fusions or both pRR1 and pPA1-pPA4 plasmids were grown at 28 °C for 24 h in M1 medium supplemented with 0.1 mM NH_4_Cl (low-ammonium conditions) or 10 mM NH_4_Cl (high-ammonium conditions) and appropriate antibiotics. In addition, Rt24.2 (pBBR1MCS-5) derivatives with pPA1-pPA4 plasmids were used as control strains. To study the effect of additional copies of *exoR* on EPS production, the pRR1 and pBBR1MCS-5 plasmids were additionally introduced into the wild-type strain Rt24.2.

### 3.6. β-Galactosidase Assay

To study the effect of ammonium on *pssA* expression, a set of transcriptional *pssA-lacZ* fusions (pPA1-pPA4) was introduced into the Rt24.2 strain by conjugation. Rt24.2 derivatives containing pPA1-pPA4 fusions were cultured for 24 h in 79CA or M1 medium supplemented with tetracycline and 0.1 or 10 mM NH_4_Cl. The β-galactosidase activity assay was carried out according to the protocol devised by Miller [69]. The reported values in Miller units are averages of three independent experiments.

### 3.7. β-Glucuronidase Assay

In order to establish the expression of flagellar and motility genes, plasmids pSVP SUM, pAVP, and pVNVP containing transcriptional fusions *rem-gusA*, *flaA-gusA*, and *visN-gusA*, respectively, were transferred using conjugation into Rt24.2 and Rt270 strains. The enzyme assays for β-glucuronidase activity were carried out according to the method devised by Miller [69] with modification described by Yost *et al.* [70].

### 3.8. EPS Isolation and Quantification

For this analysis, 5-mL cultures of Rt24.2 derivatives were grown in 79CA or M1 medium supplemented with 1% glycerol for two days at 28 °C in a rotary shaker. EPS was precipitated from culture supernatants with 4 vol. of cold 96% ethanol, centrifuged 15 min at 10,000 rpm, dissolved in deionized water and analyzed for carbohydrates according to a method described by Loewus [71]. The total sugar content was calculated as glucose equivalents.

### 3.9. Phenotype Analysis of the *pssA* Mutant Rt270 Using PM (Biolog) Test

In order to compare a phenotype of the *pssA* mutant with the wild-type strain, PM microplates (PM1, PM2A, PM3B, PM4A, and PM9, Phenotype MicroArrays™, Biolog, Hayward, CA, USA) were used, according to the manufacturer’s instruction. Based on the PM1 and PM2A microplates, the ability of utilization of different carbon sources by these strains was assayed (190 compounds, including sugars and organic acids). PM3B plates were used to establish utilization of 95 nitrogen sources, and PM4A plates 94 phosphorus and sulfur compounds, accordingly. To assess rhizobial adaptation to various stress conditions, PM9 plates were used. For this experiment, bacteria of the Rt270 and Rt24.2 strains, after growing on 79CA agar plates for 48 h, were collected and washed twice with sterile water. Final suspensions of OD_600_ = 0.12 were prepared in sterile IF-0a fluid supplemented with Dilworth’s vitamins, and 100-μL aliquots were added into each well of microplates and incubated at 28 °C up to 72 h. For PM3B and PM4A plates, 1% glycerol as a carbon source and 20 μM FeCl_3_ were additionally added. Changes of color levels in the wells were monitored at the OD_750_ at regular time intervals (24 h) using the Benchmark Plus™ microplate reader (Bio-Rad Laboratories, Hercules, CA, USA). The experiment was repeated three times.

### 3.10. Motility Assay

The assay of motility of the Rt24.2 and Rt270 strains was performed using 0.3% and 0.7% M1, 0.3% and 0.7% 79CA, and 0.3% and 0.7% TY agar media. Five microliters of bacterial suspensions of OD_600_ = 0.4 prepared in sterile water was stabbed into agar plates. The plates were incubated at 28 °C for four days, and the distance of bacterial migration from the site of injection in the agar was measured and given in millimeters. The motility assay was repeated three-fold for each strain and the medium used.

### 3.11. Isolation and Analysis of Extracellular Proteins

For analysis of extracellular proteins, the *pssA* mutant Rt270 and the wild-type strain Rt24.2 were grown at 28 °C for 48 h in 100 mL TY medium. Rhizobial cells were harvested by centrifugation at 6,000× *g* for 25 min at 4 °C. The supernatants obtained were centrifuged again in the same conditions. The proteins present in the culture supernatant were concentrated by precipitation with 10% trichloroacetic acid, as described previously [21]. The protein concentration in these fractions was determined using the Coomassie brilliant blue (G-250) dye-binding method [72] and bovine serum albumin as a standard. Extracellular proteins of the Rt24.2 and Rt270 strains were separated by electrophoresis using 12% sodium dodecyl sulphate-polyacrylamide gel (SDS-PAGE) and visualized by staining with Coomassie brilliant blue G-250 (Amresco, Solon, OH, USA).

### 3.12. Plant Experiments

Seeds of red clover (*Trifolium pratense* cv. Diana) were surface sterilized as described previously [73]. Next, the seeds were placed on plates containing nitrogen-free Fåhraeus agar [49] and incubated at 22 °C up to 48 h. Then, seedlings were placed onto slants with Fåhraeus medium, and after four-day growth they were inoculated using suspensions of the Rt24.2 and Rt270 strains of OD_600_ = 0.2 (100-μL aliquots of the suspension were used to infect each plant). The plants were grown for four weeks under natural light supplemented with artificial light (14-h day at 24 °C and 10-h night at 18 °C) in a greenhouse. Nodules emerging on clover roots were counted after each week, and 28-day plants were harvested, and wet shoot mass was estimated. The experiment was repeated three times using 20 plants for each treatment.

### 3.13. Gus Histochemistry

In order to establish nodule colonization by the Rt270 mutant and the wild-type Rt24.2, bacteria of these strains were tagged with the pJBA21Tc plasmid carrying the *gusA* gene for β-glucuronidase. Clover seedlings were inoculated with the Rt24.2 (pJBA21Tc) and Rt270 (pJBA21Tc) strains and grown up to four weeks under conditions described above. For histochemical analysis, the nodules were stained as described previously [54] using 50 mM sodium phosphate buffer (pH 7.2) containing 50 μg mL^−1^ of 5-bromo-4-chloro-3-indolyl-β-*d*-glucuronide and analyzed under a Nikon light microscope (OPTIPHOT2, Nikon, Tokyo, Japan).

### 3.14. Assays for Sensitivity to Antibiotics and Stress Factors

The sensitivity of the Rt24.2 and Rt270 strains to sodium dodecyl sulfate (SDS), sodium deoxycholate (DOC), sarcosyl, and ethanol was studied, and the minimal inhibitory concentration of the individual stressor was determined. Bacteria were collected from 79CA agar medium into sterile water to an OD_600_ of 0.2 and 10-μL aliquots of these suspensions were placed on plates containing 79CA medium and defined concentrations of SDS (0.005%–1% *w*/*v*), DOC (0.005%–1% *w*/*v*), sarcosyl (0.005%–1% *w*/*v*), and ethanol (0.005%–5% *v*/*v*). The growth of the strains on individual media was determined after four-day incubation at 28°C. The sensitivity of the Rt24.2 and Rt270 strains to antibiotics was established using commercially available filter discs with the following substances: gentamicin (10 μg), tetracycline (30 μg), chloramphenicol (30 μg), erythromycin (15 μg), neomycin (30 μg), ampicillin (10 μg), penicillin G (10 μg), bacitracin (10 μg), oxocillin (1γ), amoxicillin (2.5γ), and polimyxin B (7.5 μg) (Mast Diagnostics, Merseyside, UK). One-hundred microliter aliquots of bacterial suspensions were spread on plates containing 79CA agar, and then filter discs were placed on the surface of the medium. After four-day incubation at 28 °C, the diameter of the growth inhibition zones was measured.

## 4. Conclusions

Acidic exopolysaccharide secreted in large amounts by *R. leguminosarum* bv. *trifolii* wild-type bacteria plays a crucial role in establishment of an effective symbiosis with its host plant—*Trifolium pratense*. The synthesis of this polymer is conducted by a large multi-protein complex located in the bacterial inner membrane. PssA is one of the constituents of this enzymatic complex and initiates the process of EPS synthesis (this protein is responsible for addition of glucose-1-phosphate to the polyprenyl phosphate carrier). The *pssA* gene of *R. leguminosarum* bv. *trifolii* encodes the protein of the length of 200 aa, in which the *N*-terminus is highly hydrophobic, whereas the *C*-terminus is more hydrophilic and responsible for its enzymatic function [7,11,14,15]. In this study, we characterized the mutant strain Rt270 with a Tn*5* insertion in the 3′-end of *pssA* and indicated that the mutation in this gene caused pleiotropic effects in *R. leguminosarum* bv. *trifolii*. Several physiological defects in this *pssA* mutant were reported, among them the lack of EPS synthesis, impairment in motility and utilization of some nutrients, an altered profile of extracellular proteins, and changes in the sensitivity to several antibiotics and membrane stressors. These data confirm that the mutation of the *C*-terminus of this protein results in loss of its enzymatic activity and biological function(s) in rhizobia. This suggests that PssA is not only a key enzyme in the EPS synthesis pathway, but the presence of this protein is also important for proper functioning of bacterial cells.

## Figures and Tables

**Table 1.** Motility of the *Rhizobium leguminosarum* bv*. trifolii* wild-type and the *pssA* mutant strains assayed in different media.

Strain	Migration distance (mm) a					

	M1		79CA		TY	

	0.3%	0.7%	0.3%	0.7%	0.3%	0.7%
Rt24.2 (wild-type)	9.5 ± 1.0	3.5 ± 0.5	19 ± 2.0	6.5 ± 1.0	15 ± 2.0	4.0 ± 0.5
Rt270 (*pssA*)	4.5 ± 0.5 *	2 ± 0.5 *	11 ± 1.0 *	1.5 ± 0.5 *	7.0 ± 0.5 *	1.5 ± 0.5 *

a Migration of bacteria was determined after four-day incubation at 28 °C by measuring the distance from the injection site of bacterial suspensions into agar;

* indicates a statistically significant difference in migration zones compared to the wild-type strain (*p* value < 0.005; Student’s *t* test).

**Table 2.** The expression of *visN-gusA*, *rem-gusA*, and *flaA-gusA* fusions in the *Rhizobium leguminosarum* bv*. trifolii* wild-type and the *pssA* mutant backgrounds.

Fusion	Promoter activity in 79CA medium *			Promoter activity in M1 medium *		

	Rt24.2 (wild-type)	Rt270 (*pssA* mutant)	Ratio Rt270/Rt24.2	Rt24.2 (wild-type)	Rt270 (*pssA* mutant)	Ratio Rt270/Rt24.2
pVNVP (*visN-gusA*)	5496 ± 393 a	3021 ± 251 b	0.55	3297 ± 225 b	2326 ± 187 c	0.7
pSVP SUM (*rem-gusA*)	12707 ± 689 a	8015 ± 421 b	0.63	8640 ± 496 b	6493 ± 422 c	0.75
pAVP (*flaA-gusA*)	8804 ± 498 a	5746 ± 339 c	0.65	6855 ± 376 b	3512 ± 198 d	0.51
pFus1par (control)	131 ± 16 a	126 ± 11 a	0.96	110 ± 12 a	104 ± 15 a	0.94

* Given values in Miller units (± standard deviation) are averages of three independent experiments with three biological repetitions for each strain and treatment;

a,b,c,d indicate statistically significant differences for an individual transcriptional fusion tested in different strains and growth conditions (*p* value < 0.05; ANOVA, Tuckey’s test).

**Table 3.** Sensitivity of the wild-type *Rhizobium leguminosarum* bv. *trifolii* 24.2 and the mutant strains Rt270, MM3, and MM4 to various stressors.

Strain	Minimal inhibitory concentration *			

	SDS (% *w*/*v*)	DOC (% *w*/*v*)	Sarcosyl (% *w*/*v*)	Ethanol (% *v*/*v*)
Rt24.2 (wt)	0.025 ± 0.005 a	0.11 ± 0.005 a	0.05 ± 0.005 a	5.5 ± 0.25 a
Rt270 (*pssA*)	0.020 ± 0.005 a	0.07 ± 0.005 c	0.06 ± 0.005 a	3.5 ± 0.25 b
MM3 (*pssD*)	0.015 ± 0.005 a	0.09 ± 0.005 b	0.05 ± 0.005 a	5.0 ± 0.25 a
MM4(*pssJIHGF*)	0.015 ± 0.005 a	0.085 ± 0.005 b	0.045 ± 0.005 a	5.0 ± 0.25 a

* Given values are averages of three independent experiments with 3 biological repetitions for each strain and treatment;

a,b,c indicate statistically significant differences (in column) for an individual stress factor tested (*p* value <0.05; ANOVA, Tuckey’s test).

**Table 4.** Sensitivity of the wild-type *Rhizobium leguminosarum* bv. *trifolii* 24.2 and the *pssA* mutant Rt270 strains to various antibiotics.

Antibiotics	Growth inhibition zone (mm) *			

	Rt24.2 (wt)	Rt270 (*pssA*)	MM3 (*pssD*)	MM4 (*pssJIHGF*)
Gentamicin	28 ± 3 b	30 ± 2 b	36 ± 3 a	37 ± 3 a
Tetracycline	52 ± 4 a	49 ± 3 a	58 ± 3 a	54 ± 3 a
Chloramphenicol	54 ± 4 a	44 ± 3 b	58 ± 2 a	56 ± 4 a
Erythromycin	18 ± 2 a	12 ± 2 b	16 ± 2 a	17 ± 2 a
Neomycin	21 ± 3 a	11 ± 2 b	24 ± 2 a	26 ± 3 a
Ampicillin	12 ± 2 b	0 ± 0 c	23 ± 2 a	27 ± 2 a
Penicillin G	6 ± 1 a	0 ± 0 b	7 ± 1 a	6 ± 2 a
Amoxicillin	11 ± 2 b	0 ± 0 c	16 ± 2 a	17 ± 2 a
Bacitracin	13 ± 2 a	0 ± 0 b	10 ± 3 a	9 ± 3 a
Oxocillin	10 ± 2 a	0 ± 0 b	7 ± 1 a	6 ± 2 a
Polimyxin B	10 ± 1 a	0 ± 0 b	11 ± 2 a	12 ± 2 a

* Growth inhibition zones were determined after four-day incubation at 28 °C. Given values are averages of three independent experiments;

a,b,c indicate statistically significant differences between analyzed strains in the presence of a particular antibiotic (*p* value < 0.05; ANOVA, Tuckey’s test).

**Table 5.** The influence of multiple *exoR* copies on *pssA-lacZ* expression in different *Rhizobium leguminosarum* bv. *trifolii* 24.2 derivatives under low- and high-ammonium conditions.

Type of *pssA-lacZ* fusion	β-galactosidase activity at different NH_4_Cl concentration (Miller units) *					

	Rt24.2 (wild-type)		Rt24.2pRR1 (*exoR*)		Rt24.2 (pBBR1MCS-5)	

	0.1 mM	10 mM	0.1 mM	10 mM	0.1 mM	10 mM
pPA1	4954 ± 389 a	4804 ± 352 a	1676 ± 148 c	2286 ± 186 b	4869 ± 363 a	4617 ± 327 a
pPA2	3773 ± 293 a	3034 ± 276 b	2029 ± 178 c	2318 ± 201 c	3702 ± 311 a	2966 ± 284 b
pPA3	1878 ± 143 a	1545 ± 131 b	944 ± 89 c	1329 ± 124 b	1821 ± 139 a	1457 ± 126 b
pPA4	1540 ± 121 a	1389 ± 120 a	827 ± 76 c	1103 ± 96 b	1498 ± 132 a	1317 ± 115 a

* Given values (±standard deviation) are averages of three independent experiments with three biological repetitions for each strain and treatment;

a,b,c indicate statistically significant differences for an individual transcriptional fusion tested in different strains and growth conditions (*p* value <0.05; ANOVA, Tuckey’s test).

**Table 6.** Bacterial strains, plasmids, and oligonucleotide primers used in this study.

Strains and plasmids	Relevant characteristics	Sources or reference
***R. leguminosarum***		
Rt24.2	Wild type, Rif^r^, Nx^r^	[20]
Rt270	Rt24.2 with a mini-Tn*5* transposon insertion in 412/413 bp position of *pssA* coding region, Km^r^	This work
MM4	Rt24.2 with mTn*5*SS*gusA*40 transposon in the EPS I region (*pssJIHGF*), Sp^r^	This work
MM3 *E. coli*	Rt24.2 with mTn*5*SS*gusA*40 transposon, *pssD*, Sp^r^	This work
DH5α	*supE*44Δ*lac*U169(ϕ80*lacZ*ΔM15)*hsdR*17 *recA*1*endA*1*gyrA*96 *thi*-1 *relA*1	[60]
S17-1	294, *thi*, RP4-2-Tc::Mu-Km::Tn*7*	[63]
**Plasmids**		
pUC19	Cloning and sequencing vector, Ap^r^	[60]
pSUP202	pBR325 derivative, *mob*, Cm^r^, Tc^r^, Ap^r^	[63]
pBBR1MCS-5	*mob*, *lacZ*α, Gm^r^ cloning vector	[64]
pM34	pUC19 containing 3.4-kb *EcoR*I fragment with *pssA*	[8]
pMT27	pUC19 containing 3.4-kb *EcoR*I fragment with mini-Tn*5* in position 412/413 bp of *pssA* coding region	This work
pMSUP27	pSUP202 containing 4.6-kb *EcoR*I fragment with mini-Tn*5* inserted in 412/413 bp of *pssA* ORF	This work
pPA1	pMP220 carrying the −750 bp to +152 bp fragment of the *pssA* regulatory region, Tc^r^	[26]
pPA2	pMP220 carrying the −538 bp to +152 bp fragment of the *pssA* regulatory region, Tc^r^	[26]
pPA3	pMP220 carrying the −374 bp to +152 bp fragment of the *pssA* regulatory region, Tc^r^	[26]
pPA4	pMP220 carrying the −284 bp to +152 bp fragment of the *pssA* regulatory region, Tc^r^	[26]
pJBA21Tc	pJB321 derivative carrying constitutively expressed *gusA*, Tc^r^	[65]
pEXOR13	pUC19 containing 1.3 kb *Eco*RI fragment with Rt24.2 *exoR* gene	This work
pRR1	pBBR1MCS-5 containing 1.3 kb *Eco*RI fragment with Rt24.2 *exoR* gene	This work
pFUS1par	pMP220 derivative with promoterless *gusA*, *par*, Tc^r^	[66]
pAVP	pFUS1, *flaA::gusA*, Tc^r^	Michael Hynes, [40]
pVNVP	pFUS1P, *visN::gusA*, Tc^r^, *par* stabilized	Michael Hynes, [40]
pSVP SUM	pFUS1P, *rem::gusA*, Tc^r^, *par* stabilized	Michael Hynes, [40]
**Primers (5′-3′)**		
pExoR1	CGTTTGAATTCGGTCGTTTCGCTT	This work
pExoR3	GAACAACGGAATTCGCATCGACCA	This work

The sequences for *Eco*RI restriction sites are underlined. Nx^r^, nalidixic acid resistance; Rif^r^, rifampicin resistance; Tc^r^, tetracycline resistance; Gm^r^, gentamicin resistance; Km^r^, kanamycin resistance; Sp^r^, spectinomycin resistance.
